# Understanding the impact of bilateral brain injury in children with unilateral cerebral palsy

**DOI:** 10.1002/hbm.24978

**Published:** 2020-03-05

**Authors:** Alex M. Pagnozzi, Kerstin Pannek, Jurgen Fripp, Simona Fiori, Roslyn N. Boyd, Stephen Rose

**Affiliations:** ^1^ CSIRO Health and Biosecurity The Australian e‐Health Research Centre Brisbane Australia; ^2^ Queensland Cerebral Palsy and Rehabilitation Research Centre, Faculty of Medicine, Centre for Children's Health Research The University of Queensland Brisbane Australia; ^3^ IRCCS Stella Maris Foundation Pisa Italy

**Keywords:** bilateral, cerebral palsy, interventions, magnetic resonance imaging, plasticity

## Abstract

The presence of bilateral brain injury in patients with unilateral cerebral palsy (CP) may impact neuroplasticity in the ipsilateral hemisphere; however, this pattern of injury is typically under‐analyzed due to the lack of methods robust to severe injury. In this study, injury‐robust methods have been applied to structural brain magnetic resonance imaging (MRI) data of a cohort of 91 children with unilateral CP (37 with unilateral and 54 with bilateral brain injury, 4–17 years) and 44 typically developing controls (5–17 years), to determine how brain structure is associated with concurrent motor function, and if these associations differ between patients with unilateral or bilateral injury. Regression models were used to associate these measures with two clinical scores of hand function, with patient age, gender, brain injury laterality, and interaction effects included. Significant associations with brain structure and motor function were observed (Pearson's *r* = .494–.716), implicating several regions of the motor pathway, and demonstrating an accurate prediction of hand function from MRI, regardless of the extent of brain injury. Reduced brain volumes were observed in patients with bilateral injury, including volumes of the thalamus and corpus callosum splenium, compared to those with unilateral injury, and the healthy controls. Increases in cortical thickness in several cortical regions were observed in cohorts with unilateral and bilateral injury compared to controls, potentially suggesting neuroplasticity might be occurring in the inferior frontal gyrus and the precuneus. These findings identify prospective useful target regions for transcranial magnetic stimulation intervention.

## 1 INTRODUCTION

Cerebral palsy (CP) is a disorder of movement and posture due to damage to the developing brain during pregnancy, around birth, or in the first 28 days of life. Several comorbidities may develop in CP, such as epilepsy, intellectual disability, or visual impairment (Mutch, Alberman, Hagberg, Kodama, & Perat, 1992; Novak, Hines, Goldsmith, & Barclay, 2012; Rosenbaum, Nigel, Leviton, Goldstein, & Bax, 2007). Approximately two in every 1,000 live born children develop CP (Stanley, Blair, & Alberman, 2000), and these children often survive to adulthood, requiring life‐long interventions and management. The range of pathologies leading to CP may be severe and can affect one or both hemispheres of the brain, and even in hemiplegic (unilateral) cerebral palsy (UCP) where only one side of the body is affected, bilateral brain injury may be present. The presence of lesions in both hemispheres impacts the amount of potential cortical reorganization that may occur within patients (Kirton, 2013), especially as bilateral brain changes occur even in response to unilateral injury (Graziadio, Tomasevic, Assenza, Tecchio, & Eyre, 2012). This in turn has important implications for long‐term motor impairment (Eyre, 2007), as well as the choice of interventions in children with CP (Carr, Harrison, Evans, & Stephens, 1993; Sutcliffe, Gaetz, Logan, Cheyne, & Fehlings, 2007).

Although bilateral brain injury has been observed in approximately 30–50% of children with UCP (Feys et al., 2010; Mailleux et al., 2017; Scheck et al., 2016), little is known about the impact of bilateral brain injury on motor outcomes, especially in comparison to children with lesions localized to only one cerebral hemisphere. Upper limb function is known to be impacted by lesion location and timing (Holmefur et al., 2013; Mailleux et al., 2017); however, these studies did not find an association with bilateral lesions and bilateral hand performance, potentially as brain injuries were only measured semi‐quantitatively. Quantifications of brain structure and injury can help identify the different patterns of injury, and to isolate specific brain regions associated with motor function. Although automated approaches are necessary to produce quantitative measures without requiring manual and time‐consuming annotations on MRIs, the quantification of potentially severe bilateral brain injury in UCP cohorts would require advanced image analysis techniques, which are robust to this severe injury (Pagnozzi, Gal, et al., 2015). Using such methods, an improved understanding of the association between brain structure and motor function, particularly for patients with bilateral and potentially severe injury could be obtained, which could help to improve our understanding of cortical reorganization, and may identify potential targets for therapy via targeted transcranial magnetic stimulation (TMS; Chen et al., 2019).

Therefore in this study, quantifications of brain structure are obtained using an automated pipeline on a cohort of 91 children and adolescents with a clinical presentation of UCP (of whom 37 had unilateral injury, and 54 had bilateral brain injury), as well as 44 age‐matched typically developing controls (TDC). To investigate the differences in brain structure and motor function for patients with unilateral and bilateral brain injury, hand function was assessed using two measures, the Assisting Hand Assessment (AHA; Krumlinde‐Sundholm, Holmefur, Kottorp, & Eliasson, 2007) and the Melbourne Unilateral Upper Limb assessment (MUUL) (Bourke‐Taylor, 2007). The aim of this study is to investigate if upper limb function can be predicted from a quantitative characterization of brain structure, and if the laterality of brain injury plays an important role in this association. This may help to identify how general patterns of injury may differ between the groups, and if there are any different associations and areas of potential reorganization that are specific to patients with bilateral injury. Ultimately, this can help to identify regions associated with improved outcomes, or potential areas of compensatory mechanisms that can be a future target for therapy.

## 2 METHODS

### 2.1 Participants

Imaging and clinical data of a cohort of 91 children and adolescents presenting with UCP and 44 TDC were acquired by the Queensland Cerebral Palsy and Rehabilitation Research Centre (QCPRRC) as part of the Move It To Improve It (Mitii) study (Boyd, Mitchell, et al., 2013), which was registered with the Australian clinical trials register (ACTRN12611001174976). Participants were identified through a population‐based research database, comprising over 1,600 children and adolescents with UCP. Of the children and adolescents with a clinical presentation of UCP, 37 had unilateral injury and 54 had bilateral brain injury. Scans from an age‐matched TDC (*n* = 44) were included. Diagnosis of UCP was confirmed by a pediatrician or clinician in accordance with published recommendations (Badawi et al., 2008). As Mitii is an intervention trial, only baseline data were used in this study. This trial included children and adolescents with spastic‐type mild to moderate congenital hemiplegia aged 8–18 years recruited across Queensland and New South Wales, who are Gross Motor Function Classification (GMFCS) I or II with sufficient cooperation to perform the tasks. Participants were excluded if they had received upper or lower limb surgeries in the previous 6 months, had unstable epilepsy, or a respiratory, cardiovascular or other medical condition that would prevent safe participation in the Mitii training. Written informed consent was obtained from the parent or legal guardian of each child. Ethical approval was obtained by the Medical Ethics Committee of The University of Queensland (2011000608), The Royal Children's Hospital Brisbane (HREC/11/QRCH/35), and the Cerebral Palsy Alliance Ethics Committee (April 1, 2013). The data that support the findings of this study are available on request from the corresponding author. The data are not publicly available due to privacy or ethical restrictions.

### 2.2 Image acquisition

For all participants T1 Magnetization Prepared Rapid Gradient Echo (MPRAGE) was acquired (*n* = 139) on one of two different scanners, including a 3T Siemens MAGNETOM Trio Tim scanner with scanning parameters (TR = 1,900 ms, TE = 2.32 ms, TI = 900 ms, flip angle = 9°, slice thickness = 0.9 mm), or a 1.5 T GE Genesis SIGNA scanner with two different scanning parameters (TR = 14.28 ms, TE = 4.36 ms, TI = 700 ms, flip angle = 13°, slice thickness = 1 mm) and (TR = 12.4 ms, TE = 2.39 ms, TI = 700 ms, flip angle = 10°, slice thickness = 1 mm). This slight variation in image sequences necessitates the developed image processing algorithms to be robust to the applied protocol. In the same session, a subset of patients (*n* = 104) also underwent either axial T2 Turbo Inversion Recovery Magnitude (TIRM; TR = 7,000 ms, TE = 79 ms, flip angle = 120°, slice thickness = 4 mm) or T2 Half‐Fourier Acquisition Single‐Shot Turbo Spin‐Echo (HASTE; TR = 1,500 ms, TE = 81 ms, flip angle = 150°, slice thickness = 4 mm), which was acquired solely using the 3T Siemens scanner.

### 2.3 Semi‐quantitative scoring

The acquired MR images were scored with a semi‐quantitative brain lesion severity scale (Fiori et al., 2014) by a single child neurologist with training in brain MRI (SF), using the T1‐weighted and T2 fluid‐attenuated inversion recovery (TIRM) MR images of the same patient. In this scoring technique, observed tissue abnormality was manually drawn onto six representative slices from the brain in the cerebral lobes (temporal, frontal, parietal, and occipital), subcortical structures (e.g., basal ganglia, thalami, brainstem, and posterior limb of the internal capsule [PLIC]), corpus callosum, and cerebellum. Each region except the corpus callosum is scored independently for both hemispheres, either “0” or “1” if not impacted or impacted, respectively. Using this scoring approach, lesion laterality was defined as a categorical variable in this study, including “no injury” which consisted of TDCs who were not scored using the semi‐quantitative scale approach, “unilateral” were patients with UCP and who had an injury score of 0 in one hemisphere, and “bilateral injury” where injury was present on both hemispheres. No participants diagnosed with UCP were scored as having “no injury” using this template.

### 2.4 Image segmentation

**Figure 1:**
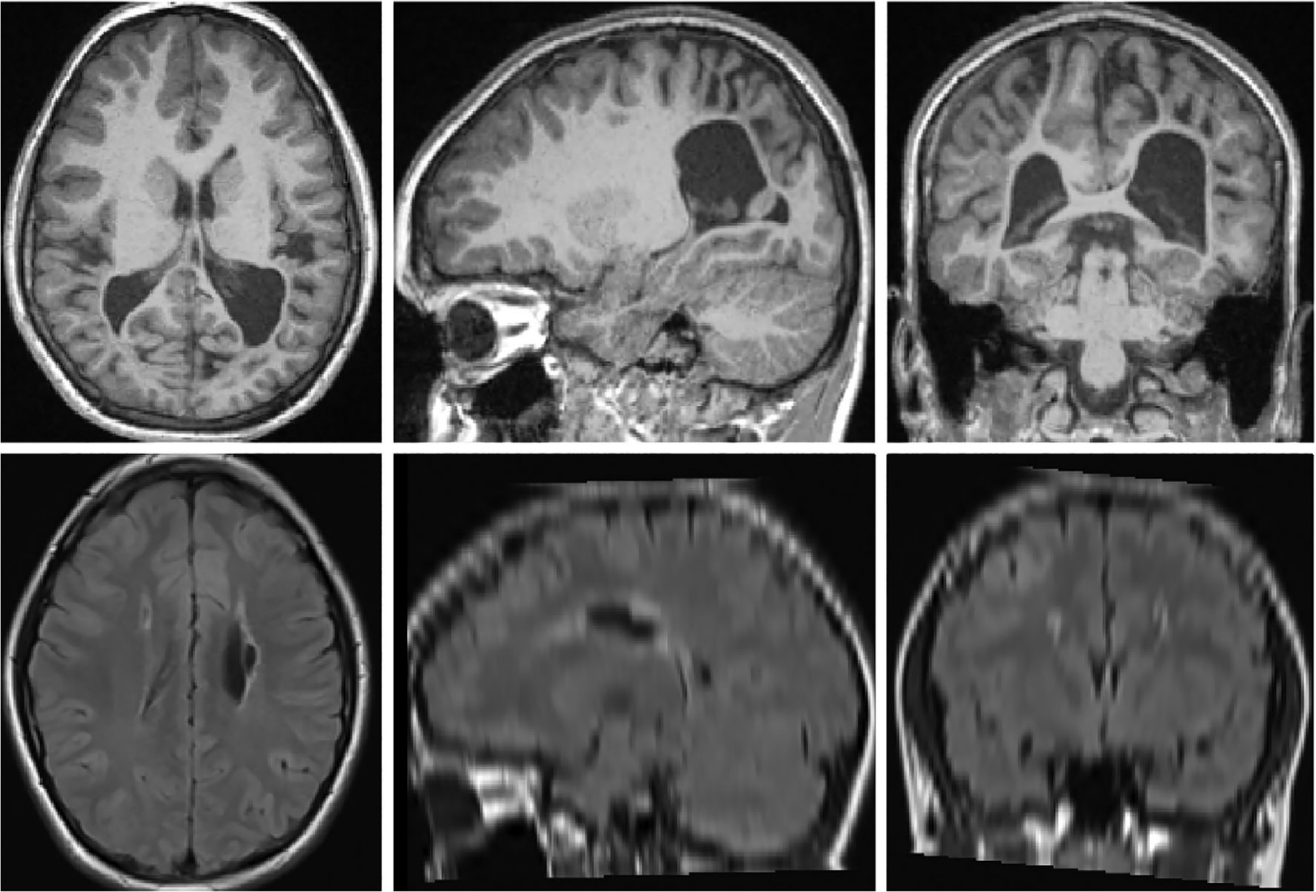
Axial (first column), sagittal (second column) and coronal views (third column) from the T1 and T2 weighted MRIs of two participants with CP classified with bilateral injury. Row 1 shows a participant with bilaterally enlarged ventricles and posterior cortical atrophy. Row 2 shows a participant with bilateral periventricular leukomalacia, visible as the hyperintense regions from the T2 TIRM sequence. Note that this sequence has a large slice thickness (5.2 mm), the resampled isotropic image appears blurry in both sagittal and coronal views. CP, cerebral palsy; MRI, magnetic resonance image; TIRM, turbo inversion recovery magnitude

Due to the presence of severe unilateral and bilateral injury in this UCP cohort, illustrated with two participants in Figure 1, attention must be paid to the accuracy of automated algorithms. For instance, with the two MRIs shown in Figure 1, the extensive injury would impact atlas‐based approaches for tissue segmentation as the image differs substantially from prior maps of anatomy. Therefore, this study utilized injury‐robust approaches that have been developed previously and validated on a UCP cohort. These methods include tissue segmentation, allowing measures of tissue volume to be computed (white matter, gray matter, lateral ventricles, deep gray matter, corpus callosum), as well as measures of cortical shape and lesion volumes (Pagnozzi, Dowson, et al., 2015; Pagnozzi, Dowson, Doecke, et al., 2016; Pagnozzi, Dowson, Fiori, et al., 2016).

#### 2.4.1 Image preprocessing

**Figure 2:**
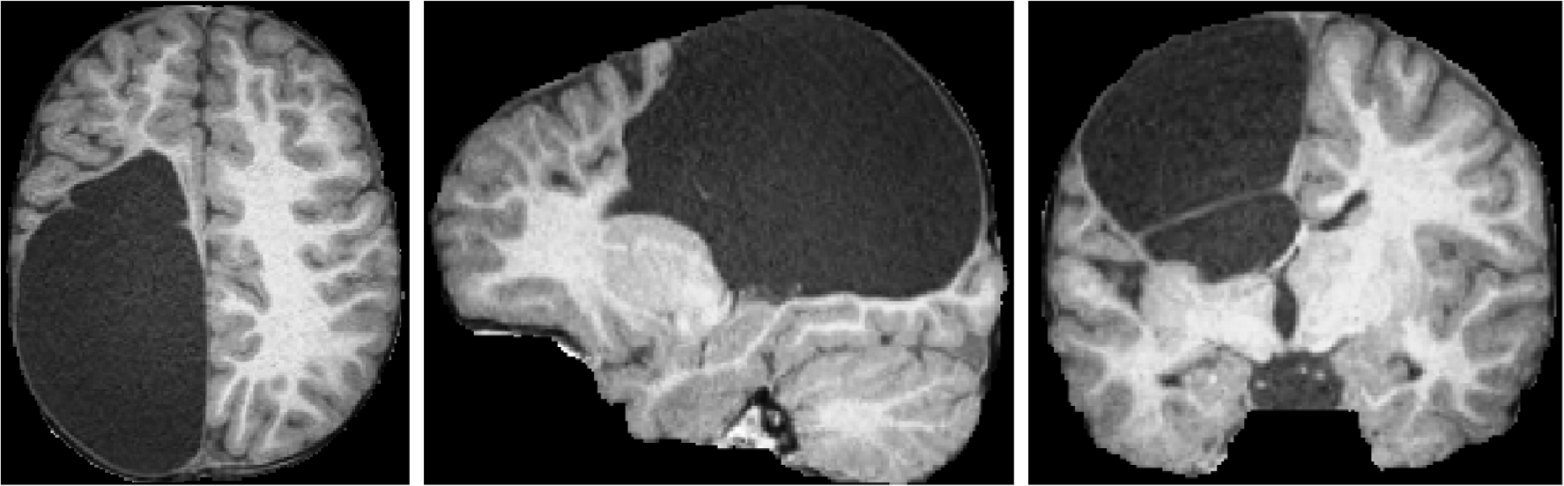
Illustration of the brain extraction pre‐processing step, of a study participant with CP and a large unilateral lesion. CP, cerebral palsy

MRI preprocessing steps were performed on both MPRAGE and TIRM sequences, which included N4 bias correction (Tustison et al., 2010), histogram equalization using the InsightToolkit (ITK), image denoising using anisotropic diffusion (Perona & Malik, 1990) and affine alignment to the Colin 27 Average Brain Atlas (The McConnell Brain Imaging Centre, 2012) using Advanced Normalization Tools registration algorithm (Rivest‐Hénault, Dowson, Greer, Fripp, & Dowling, 2015). This affine alignment essentially normalized the intracranial volumes across study participants. Skull stripping was performed in the atlas space using an in‐house algorithm that estimates white matter intensity from sampling the center of gravity of the image, obtaining a naïve WM segmentation, and extending the brain mask until intradural cerebrospinal fluid (CSF) is reached (i.e., a voxel intensity less than 25% of the naïve WM intensity is observed). This approach is capable of accurately segmenting the brain in cases of large lesions (Pagnozzi, Dowson, Doecke, et al., 2016), as illustrated in Figure 2. T2‐weighted MRI were also pre‐processed with N4 bias correction, anisotropic diffusion denoising, alignment to the Colin 27 atlas by first aligning it to the raw T1, and then applying the affine transform obtained from the T1 MRI alignment, and brain masking using the T1 MRI mask.

#### 2.4.2 Tissue segmentation

**Figure 3:**
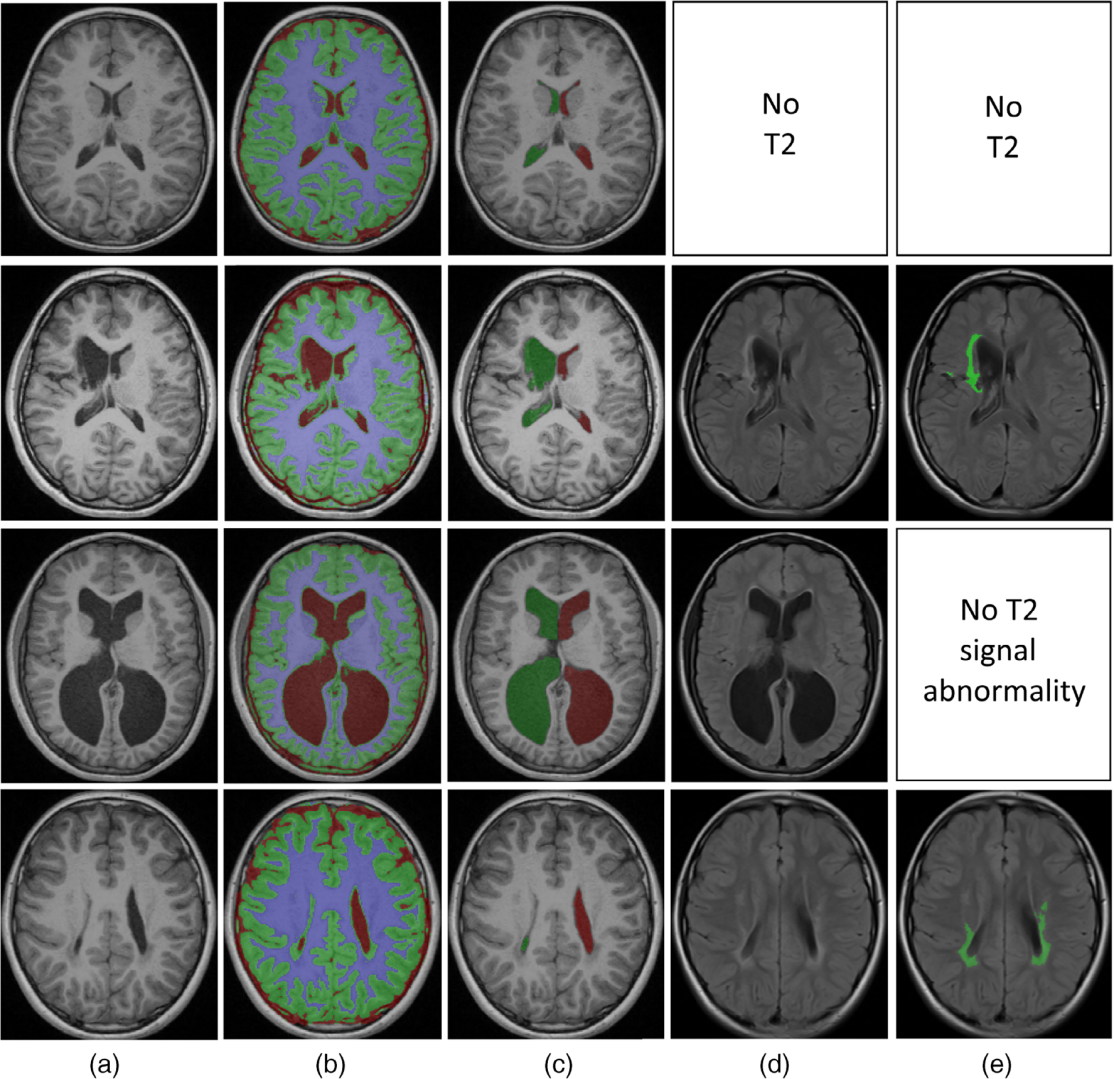
Illustrations of several tissue segmentations from four study participants. (a) Shows the pre‐processed T1‐MRI, (b) shows the WM, GM and CSF segmentations, (c) Shows the left and right lateral ventricle segmentations, (d) shows the pre‐processed T2‐MRI and (e) shows the obtained lesion segmentation. These participants were chosen to illustrate the common injury types in this cohort (Rows 2 and 3—enlarged ventricles, Rows 2 and 4—white matter lesions) and the differing availability of the T2 sequence (Row 1—no T2 MRI was available and so no lesion segmentation was performed, while in Row 3 lesion segmentation was performed and no T2 signal abnormality was found). CSF, cerebrospinal fluid; GM, gray matter; MRI, magnetic resonance image; WM, white matter

Several automated measures of brain structure were obtained on using pathology‐robust approaches, with reduced reliance on a priori information, to identify tissue volumes (i.e., WM, GM, CSF; Pagnozzi, Dowson, et al., 2015; Zhang, Brady, & Smith, 2001), and lesion volumes in the WM and GM (Pagnozzi, Dowson, Doecke, et al., 2016). These approaches can quantify any potential tissue loss resulting from hypoxic–ischemic events (HIE) and periventricular leukomalacia (PVL), which are both common forms of injury in children and adolescents with UCP (Korzeniewski, Birbeck, DeLano, Potchen, & Paneth, 2008). As ventricular enlargement may occur, either as a secondary form of injury to cystic PVL or due to hemorrhage, the volumes of the lateral ventricles were segmented from the CSF segmentation (Nosarti et al., 2002). Finally the presence of WM and GM lesions were segmented using the Expectation Maximization algorithm, and utilized a T2‐TIRM sequence in which these lesions appear hyperintense, to weight membership into a fourth lesion class (in addition to the GM/WM/CSF healthy tissue classes) (Pagnozzi, Dowson, Doecke, et al., 2016). Examples of these segmentations are illustrated in Figure 3.

#### 2.4.3 Cortical region delineation

To detect a range of cortical shape abnormalities resulting from the presence of lesions (Barkovich, 2002), several measures of cortical shape are computed from the cortical GM segmentation, including cortical thickness (CT), sulcal depth (SD), and cortical curvature (Pagnozzi, Dowson, Fiori, et al., 2016). Cortical labels were propagated from the Automated Anatomical Labeling (AAL) atlas to identify cortical shape in different areas of the cortex. This approach propagates labels for larger distances in cases of severe cortical deformities/abnormalities, however, like other structural approaches, segments cortical areas based on structural organization rather than functional organization. Hence, the resulting labels may not accurately represent the underlying function in cases of reorganization.

#### 2.4.4 Deep gray matter, internal capsule, and corpus callosum segmentation

**Figure 4:**
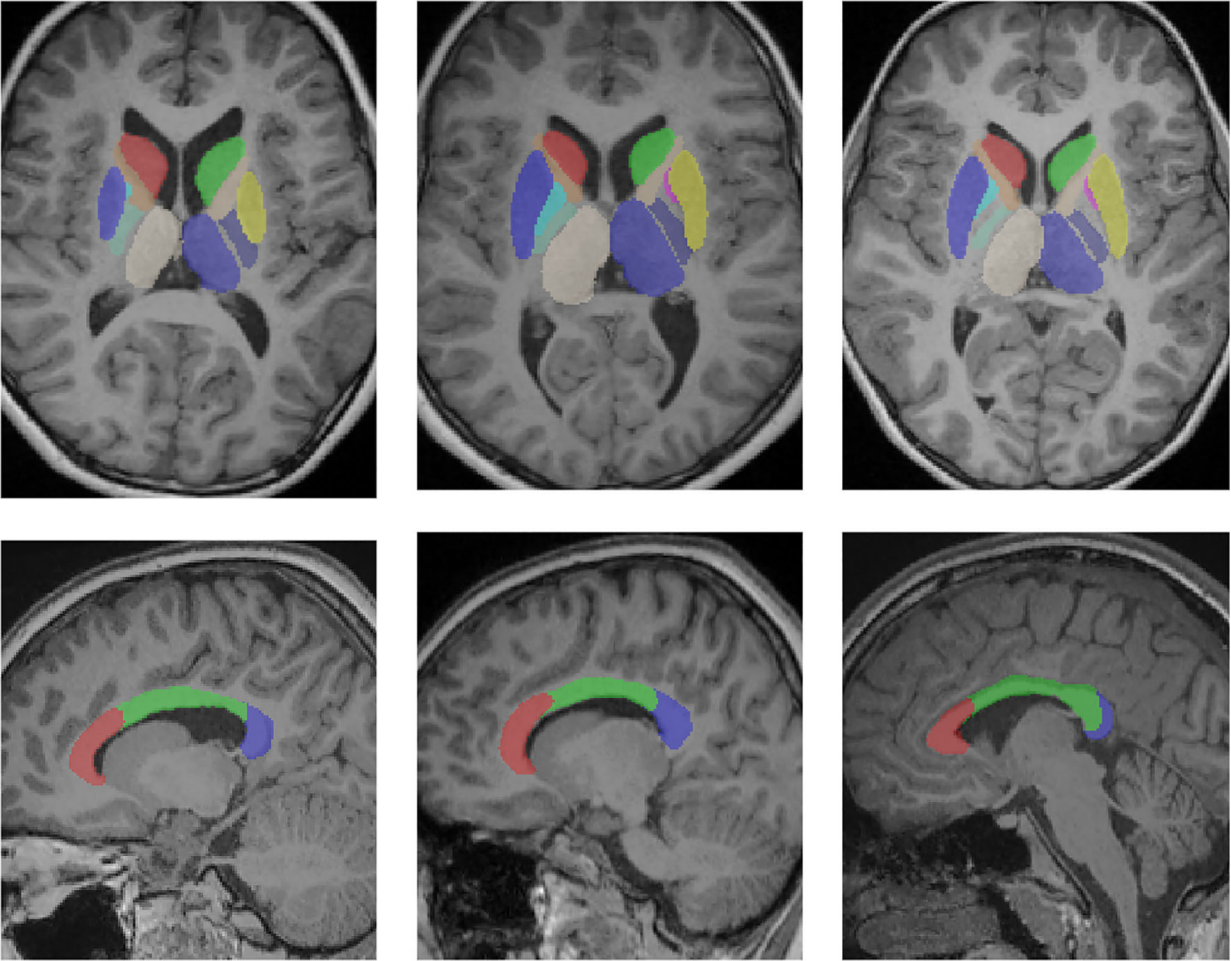
Illustration of the deep gray matter and internal capsule labels (top row) and corpus callosum segments (bottom row) in patients with CP. CP, cerebral palsy

In addition to the volumetric variables measured above, the volumes of the deep gray matter (DGM) structures (including the caudate nucleus, lenticular nucleus, and the thalamus), the anterior and posterior limbs of the internal capsule as well as the genu, body and splenium of the corpus callosum were also computed. The DGM labels were obtained from AAL atlas, while the internal capsule and corpus callosum labels were obtained from the International Consortium of Brain Mapping white matter atlas. Histogram equalization of the brain masked T1 was performed to match to the Colin 27 atlas. Then the DGM, internal capsule, and corpus callosum were rigidly aligned to the T1 to optimally border the lateral ventricles (with the corpus callosum aligned to superior to lateral ventricles, and the DGM aligned lateral to lateral ventricles). This was performed as the ventricle position may not be in the center of the skull due to severe tissue loss. Then, three‐dimensional patches of the atlas containing parts of the DGM/corpus callosum label were extracted and compared to patches from the histogram‐matched T1 using the sum of squared distance (SSD). To remove the impact of potential tissue loss, the labeled CSF in both atlas and target patches was masked out, removing it from the SSD calculation. The patch size was set to 15 × 15 × 15 voxels, and the search radius was six voxels in all three dimensions. Atlas labels were then propagated to the target patch with the lowest SSD, and summed to produce a probabilistic map of each anatomy. Thresholding was performed to provide a final segmentation, followed by postprocessing to mask out lesion segmentations obtained earlier in the pipeline. Examples of DGM, internal capsule and corpus callosum segmentations are shown in Figure 4. All segmentations were visually inspected to ensure there was not any failure in pre‐processing, segmentation, or parcellation.

### 2.5 Clinical measures

As part of the ongoing studies on children and adolescents with UCP (Boyd, Mitchell, et al., 2013; Boyd, Ziviani, et al., 2013), multiple clinical scores of function were measured by experts to provide an overview of patient function. In this study, two clinical scores were utilized to represent patient motor function. One of these is the AHA, which is a reliable measure of how well the nondominant hand is used as an assisting hand by the patient in bimanual tasks (Krumlinde‐Sundholm et al., 2007). The AHA score ranges between 0 and 100, with higher scores indicating an improved manual capability of the nondominant hand. In comparison, the MUUL assessment is a reliable measure of the quality of nondominant hand movement in unimanual tasks (Randall, Carlin, Chondros, & Reddihough, 2001), with a score from 0 to 122 reported as a percentage. Scores of gross motor and hand function were also measured, using the Gross Motor Function Classification System (GMFCS) and Manual Ability Classification System (MACS), respectively. Clinical assessments and neuroimaging were performed the same day at the QCPRRC.

### 2.6 Statistical methodology

Two random forest regression models were constructed, using quantitative measures of tissue volume, cortical shape, and lesion volumes (if T2‐TIRM was available) on the ipsilateral and contralateral hemispheres as independent variables, patient age and gender as covariates, with the AHA and MUUL measures as the outcome variable respectively. Random forests were chosen as the construction of multiple decision trees utilizing a subset of variables and data helps the model to prevent overfitting, as well as reliance on an individual measure of brain structure. In addition, lesion laterality was included as a covariate based on the semi‐quantitative scale score, which was defined as a categorical variable; “unilateral injury” or “bilateral injury.” Furthermore, to identify specific patterns of brain injury distinct to each sub‐group, interactions between the lesion laterality variable and the quantitative measures of brain structure were included in these models. Frequency of participant gender, injury etiology, injury laterality, and GMFCS and MACS scores were compared between unilateral and bilateral cohorts using a Chi‐squared test. As the TDC cohort do not have clinical measures recorded, they were excluded from the regression models.

Due to the low numbers of participants (*n* = 91 in total) compared to the large number of variables, two methods of feature reduction were implemented independently to minimize model overfitting. These include data‐driven variable selection which automatically drops variables that do not explain a sufficient amount of variance in the outcome, as well as principal component analysis (PCA) which reduces the dimensions of the data into orthogonal components (Song, Guo, & Mei, 2010). The more impactful variables of the largest eigenvectors provide an indication of which sets of variables co‐vary, and which capture most of the variance in measures observed in the cohort. The top 10 eigenvectors were retained following PCA, which contained >99% of the variance in the structural measures. Using the set of variables obtained from PCA, as well as a set of variables derived from automated step‐wise selection using the default parameters for sparsity of the random forest regressor in the “sci‐kit learn” library in Python, random forest regression was performed on a 75% training partition of the data. The performance of both models in predicting motor function was tested independently on the remaining unseen 25% using the correlation coefficient between actual and predicted motor scores. As a result, four random forests models were constructed in total (two with AHA as the outcome, two with MUUL as the outcome, each with data‐driven or PCA‐driven variable selection). Variable selection was performed on the automatically quantified measures of brain structure, demographic covariates, and the interaction variables of brain structure with the lesion laterality covariate.

Based on the retained predictor and interaction variables from the random forest, Student's *t*‐tests were performed to compare the measures of brain structure between the TDC and unilateral and bilateral cohorts, to investigate the directionality of volume differences. Distributions of structural measures were checked for normality, to ensure parametric tests were appropriate. Bonferroni correction was performed based on the number of *t*‐tests to correct for multiple comparisons.

## 3 RESULTS

### 3.1 Participant demographics

The detailed demographics and clinical characteristics of the cohort are provided in Table 1. The cohort is divided into three groups, those who do not have UCP (i.e., TDC), children and adolescents with UCP and with brain injury in only one hemisphere, according to the semi‐quantitative scale (Fiori et al., 2014) (“unilateral injury”), and children and adolescents with UCP and brain injury present in both hemispheres according to the semi‐quantitative scale (“bilateral injury”). Student's *t*‐tests were performed to investigate any demographic differences between patients with unilateral and bilateral injury, including age and measured motor function, while Chi‐squared tests were similarly performed to compare gender, etiology (periventricular white matter injury [PWM] or cortical and deep gray matter [CDGM] injury) and laterality of injury (predominantly left vs. right), and the ordinal GMFCS and MACS comparison were performed. These unilateral and bilateral brain injury cohorts were found to be comparable in both age (*p* = .871) and gender (*p* = .235), and did not show any significant difference in motor function between the two cohorts. As expected, the semi‐quantitative score of brain injury was significantly higher in the cohort with bilateral brain injury using the Student's *t*‐test (*p* < 3.86e‐07).

**Table 1:** Outline of participants demographics, as well as clinical function, of the three groups within the cohort. Statistical tests were between the unilateral and bilateral brain injury cohorts only

Cohort	TDC cohort	CP cohort (unilateral injury)	CP cohort (bilateral injury)	*p* value (unilateral vs. bilateral)
Number of participants	48	37	54	
Gender				
Male	20	24	27	
Female	28	13	27	.235
Hemiplegia laterality				
Left	NA	16	25	
Right	NA	21	29	.942
Timing of brain injury				
PWM	NA	28	39	
CDGM	NA	9	15	.901
Age at scan (years)				
Mean ± *SD*	9.2 ± 3.5	11.3 ± 3.4	11.4 ± 2.9	.871
Range (minimum–maximum)	2.2–16.9	5.4–17.4	4.1–17.4	
Global brain injury severity score (Fiori et al., 2014)				
Mean ± *SD*	NA	5.9 ± 3.2	11.0 ± 4.9	3.68e‐07
Range (minimum–maximum)	NA	2–14	2–21	
Assisting hand assessment (AHA) score				
Mean ± *SD*	NA	68.0 ± 20.7	62.2 ± 21.2	.216
Range (minimum–maximum)	NA	8.0–98.8	24.0–98.0	
Melbourne unilateral upper limb assessment (MUUL) score				
Mean ± *SD*	NA	80.5 ± 18.1	76.0 ± 20.5	.301
Range (minimum–maximum)	NA	25.0–100.0	26.2–100.0	
Manual ability classification system (MACS) score				
Mean ± *SD*	NA	1.53 ± 0.50	1.67 ± 0.47	.208
Range (minimum ‐ maximum)	NA	1.0–2.0	1.0–2.0	
Level 1	NA	19	23	
Level 2	NA	18	34	
Level 3	NA	0	0	
Level I AHA mean ± *SD*	NA	77.1 ± 33.8	78.7 ± 15.8	.859
Level II AHA mean ± *SD*	NA	87.5 ± 22.9	71.6 ± 38.5	.144
Level I MUUL mean ± *SD*	NA	63.2 ± 18.3	73.7 ± 15.8	.133
Level II MUUL mean ± *SD*	NA	65.4 ± 21.2	62.4 ± 23.1	.657
Gross motor function classification system (GMFCS) score				
Mean ± *SD*	NA	1.38 ± 0.49	1.39 ± 0.49	.929
Range (minimum–maximum)	NA	1.0–2.0	1.0–2.0	
Level 1	NA	24	37	
Level 2	NA	13	20	
Level 3	NA	0	0	

Abbreviations: AHA, Assisting Hand Assessment; CDGM, cortical and deep gray matter; CP, cerebral palsy; GMFCS, Gross Motor Function Classification System; MUUL, Melbourne Unilateral Upper Limb assessment; PWM, periventricular white matter; TDC, typically developing controls.

In addition, as the timing of the brain lesion may impact upper limb function (Mailleux et al., 2017), the ratio of PWM/CDGM injuries between unilateral and bilateral cohorts were compared, and were found to be statistically similar (*p* = .90). It was surprising to observe more participants with CDGM injury in the bilateral cohort, given the presumed mechanisms of stroke, however, the cohort allocation was only based on the MRI findings and not the presumed etiology. Therefore, even if the resulting injury was predominantly unilateral, even if subtle injury was scored on the less‐affected hemisphere than it would be classed as “bilateral” brain injury. Finally, it was observed that the ratio of MACS I and II patients differed between unilateral and bilateral cohorts, albeit not significantly (*p* = .10), and so AHA and MUUL scores were compared between MACS levels I and II, which also revealed no significant differences (*p* > .13).

### 3.2 Model construction

The cohort consisting of the combined unilateral and bilateral groups defined in Table 1, as well as patient age and gender as covariates was accumulated, and random forest models were constructed associating measures of brain structure with AHA and MUUL. The features retained using data‐driven variable selection as well as the *R* ^2^ of the models on the training set are shown in Table S1. Both models showed significant association of these features to both AHA and MUUL (*R* ^2^ of .697 and .522, respectively). Interestingly, no covariates were retained in either model, indicating that patient age, gender, or cohort (unilateral or bilateral brain injury) were not associated with motor function.

Looking at the structural features specifically, features were separated as contralateral or ipsilateral to the side of impairment. Due to the cross laterality of the brain, the contralateral side is the side of injury in the unilateral injury cohort, and likely the side of greater injury in the bilateral cohort. Most of the retained variables were isolated to the contralateral hemisphere, reflecting the degree of injury occurring on this hemisphere. Specifically, several contralateral areas with known motor roles were retained, including volume of the thalamus, PLIC, supplementary motor area, and the precentral gyrus. Features that were shared across both AHA and MUUL models include contralateral ALIC volume, CT of the contralateral inferior frontal gyrus and ipsilateral ventricle volume. Several interactions between measures of brain structure and the lesion laterality cohort were also retained in these models, which highlight regions where the difference in structure between unilateral and bilateral cohorts was significantly associated with motor function. Key differences in contralateral WM, ALIC, and PLIC volumes were retained, reflecting different severities of injuries at these locations between the two groups.

PCA was also performed as an alternative feature strategy. Among the top five features in the largest five components, frequently retained features include WM, thalamic and ventricle volumes (ipsi‐ and contralateral), contralateral GM and caudate volumes, contralateral cerebellar WM volume and the splenium of the corpus callosum, as well as interactions of the ipsi‐ and contralateral ventricle, ipsi‐ and contralateral WM, ipsi‐ and contralateral thalamus, contralateral cerebellar WM, contralateral PLIC, ipsi‐ and contralateral GM, contralateral cerebellar GM with the injury laterality cohort. The features retained using PCA also showed significant associations with both AHA and MUUL on the training set (*R* ^2^ of .570 and .480, respectively). Moreover, the smaller pool of features meant that there was a large overlap in features present in both models, as shown in Table S2. As with the data‐driven models, most covariates were not retained in either model, except for participant age in the PCA‐driven MUUL model. Similar to the data‐driven models, ipsilateral ventricle volume was retained in both PCA models, as well as splenium of the corpus callosum. Among the retained interaction variables, GM volume was the only ipsilateral feature, with all remaining interactions occurring on the contralateral side (including contralateral WM, GM, thalamus, and PLIC volumes).

**Figure 5:**
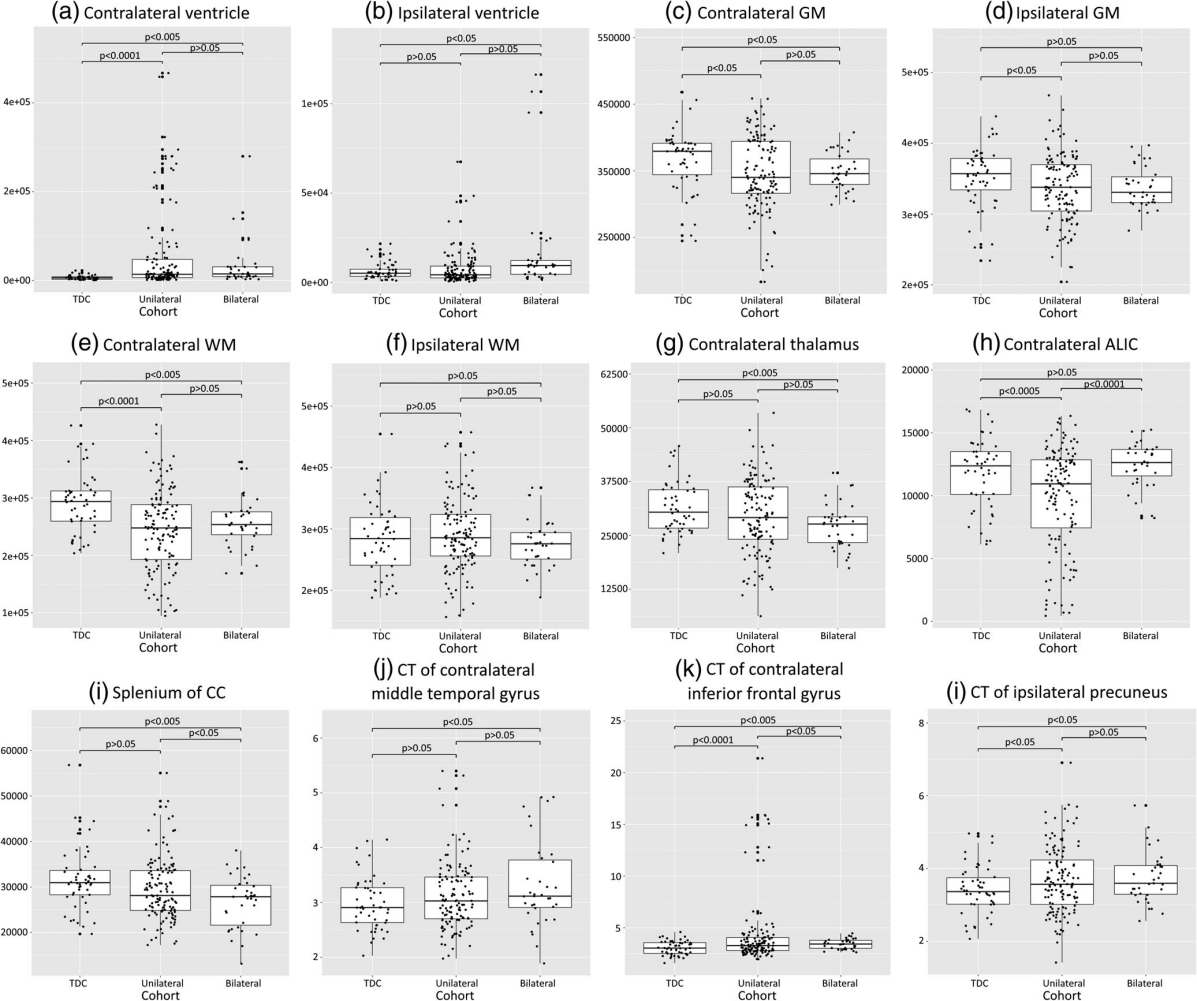
Bar charts illustrating brain measures of the TDC, unilateral and bilateral brain injury cohorts, with differences between cohorts compared using Student's *t*‐test (corrected for multiple comparisons). ALIC, anterior limb of the internal capsule; CC, corpus callosum; CT, cortical thickness; GM, gray matter; TDC, typically developing cohort; WM, white matter

Although both feature selection methods identify significant interactions between brain structure and the laterality of brain injury within the random forest models, random forests do not provide information on the direction these measures differ between cohorts. The directionality of several features are of interest, specifically the volume features largely retained by PCA feature selection, as well as local cortical features retained in both data‐driven AHA and MUUL models (which include the contralateral middle temporal gyrus, contralateral inferior frontal gyrus, and the ipsilateral precuneus). To investigate the directionality of difference in these significant features, structural measures of each cohort were compared using Student's *t*‐test, correcting for multiple comparisons. These tests revealed overall increases in ventricle volume on the contralateral side (for both unilateral and bilateral cohorts) and ipsilateral side (only on the bilateral cohort), reduced WM and GM volume on the affected, contralateral side. Reduced contralateral thalamus volumes were also detected in the unilateral cohort, and reduced contralateral ALIC and corpus callosum splenium volumes were observed in the bilateral cohort. Differences in cortical morphology revealed increased CT in the contralateral inferior frontal gyrus and ipsilateral precuneus in both unilateral and bilateral cohorts. These mean measures and the *p*‐value of the Student's *t*‐test between cohorts are provided in Table 2, and box plots for the measurements with significant cohort differences are illustrated in Figure 5. For the TDC cohort, ipsilateral and contralateral are defined as the left and right hemispheres respectively, as both are expected to be anatomically similar.

**Table 2:** Group average and *SD* of brain measures for the three cohorts, and the corrected p‐value of the corresponding group differences. Note all these measures are normalized to total intracranial volume. *p* Values are corrected for multiple comparisons using Bonferroni correction

	TDC cohort		CP cohort (unilateral injury)		CP cohort (bilateral injury)		*p* Value (TDC vs. unilateral)	*p* Value (TDC vs. bilateral)	*p* value (unilateral vs. bilateral)
	Mean	*SD*	Mean	*SD*	Mean	*SD*			
Contralateral ventricle volume (ml)	6.23	4.66	55.47	94.45	35.53	55.06	7.46 e‐08	.008	.235
Ipsilateral ventricle volume (ml)	6.43	4.58	7.70	9.57	17.48	27.91	.442	.056	.107
Contralateral GM volume (ml)	367.74	44.34	346.79	54.91	348.62	27.28	.020	.034	.157
Ipsilateral GM volume (ml)	352.89	41.71	337.03	47.63	338.04	28.34	.065	.113	.175
Contralateral WM volume (ml)	292.96	38.80	258.53	71.44	267.36	30.93	1.17 e‐05	.003	.403
Ipsilateral WM volume (ml)	286.62	42.88	292.73	44.13	281.01	28.47	.806	.952	.130
Contralateral thalamus volume (ml)	31.45	5.97	29.33	8.85	27.16	5.12	.141	.002	.136
Contralateral ALIC volume (ml)	11.84	2.76	9.88	4.05	12.50	1.73	7.00 e‐04	.365	2.14 e‐07
Contralateral PLIC volume (ml)	12.42	4.48	12.09	5.62	13.98	3.92	.138	.192	.054
CC splenium volume (ml)	31.59	3.37	29.57	3.42	26.69	2.81	.159	.001	.027
CT of contralateral inferior frontal gyrus (mm)	3.06	0.66	4.30	0.93	3.43	0.47	1.97 e‐04	.008	.010
CT of contralateral middle temporal gyrus (mm)	2.98	0.48	3.11	0.61	3.33	0.77	.288	.053	.290
CT of ipsilateral precuneus (mm)	3.40	0.66	3.69	0.92	3.74	0.69	.046	.049	.138

Abbreviations: ALIC, anterior limb of the internal capsule; CT, cortical thickness; PLIC, posterior limb of the internal capsule; SD, sulcal depth.

### 3.3 Model validation

**Figure 6:**
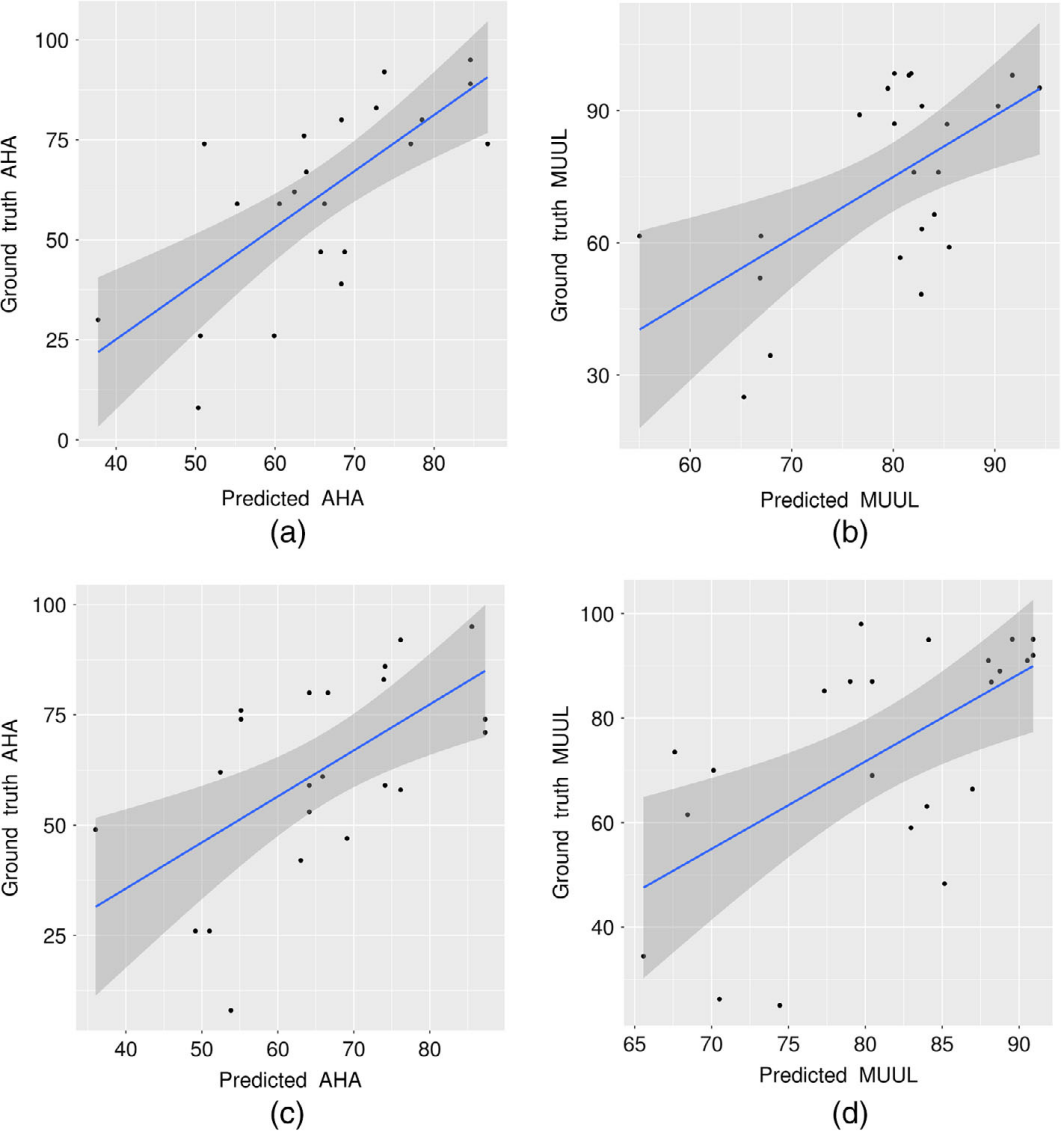
Scatter plots illustrating the correlation between model predicted motor function and the ground truth motor function on the test set for the (a) data‐driven AHA model, (b) data‐driven MUUL model, (c) PCA‐driven AHA model, and (d) PCA‐driven MUUL model. AHA, Assisting Hand Assessment; MUUL, Melbourne Unilateral Upper Limb assessment; PCA, principal component analysis

The test set performance of all constructed random forest models are provided in Table 3. Regardless of feature selection method, significant test set correlations were observed for both AHA and MUUL models as shown in Figure 6, illustrating that generalizable relationships between brain structure and motor function were identified. Comparing data‐driven and PCA selection showed that data‐driven approaches improved the prediction of AHA, while PCA was slightly better at predicting MUUL. These differences arise from the different set of features retained by each method (Table S1 and S2), with PCA retaining more global volume measures and data‐driven feature selection retained more local cortical areas.

**Table 3:** Test set correlations of all constructed random forest models

	AHA			MUUL		
	Training *R* ^2^	Test Pearson's *r*	Test RMSE	Training *R*	Pearson's *r*	Test RMSE
Data‐driven feature selection	.835	.716***	16.23	.722	.494**	18.03
PCA feature selection	.755	.654***	17.89	.693	.502**	18.22

*Note:* Asterisked feature correlations were found to be statistically significant: * *p* < .05; ** *p* < .01, *** *p* < .001.

Abbreviations: AHA, Assisting Hand Assessment; MUUL, Melbourne Unilateral Upper Limb assessment; PCA, principal component analysis; RMSE, root mean square error.

## 4 DISCUSSION

In this study, automated quantification of brain MRI was used to investigate the impact of bilateral brain injury on motor function in children and adolescents with UCP. It was observed that AHA and MUUL scores between children with unilateral and bilateral brain injury were not statistically significantly different (Table 1), despite lesions being more severe based on the semi‐quantitative scoring. This result is consistent with previous findings that the presence of unilateral or bilateral damage had no predictive value on hand performance (Holmefur et al., 2013; Mailleux et al., 2017). Furthermore, bilateral brain injury being thought to have a protective effect with respect to brain reorganization, by preventing reorganization towards the dominant hemisphere that is known to lead to poorer functional outcomes (Eyre et al., 2007).

Although the presence of bilateral brain injury did not significantly affect motor performance in the present cohort, significant differences in associations between brain structure and motor function were observed, suggesting different underlying patterns of injury between the cohorts. These differences are reflected by the significant interactions between brain structure with brain injury cohort shown in Tables S1 and S2, which for PCA consisted of WM, GM, and ventricle volumes, reflecting the destructive impact of lesion leading to the reduced brain volumes and increased CSF volumes in the affected hemisphere(s) (Figure 5). Furthermore, reduced thalamus and corpus callosum splenium volume were observed specifically in the bilateral brain injury cohort, both of which are regions associated with motor (Sommer, 2003) and visuomotor function (Rondot, de Recondo, & Dumas, 1977) respectively. In contrast for the unilateral brain injury cohort, significant reductions in the contralateral ALIC and PLIC were observed, the latter having a known role in motor function (Rose, Guzzetta, Pannek, & Boyd, 2011) and is in agreement with the known role of the PLIC in predicting hemiplegia since birth (De Vries et al., 1999; Mercuri et al., 1999). These findings suggest that focal lesions impacting the corticospinal tract were more frequent among unilateral injuries, whereas bilateral injuries were more frequently associated with global tissue loss and hence may impact other regions of the corticospinal tract, such as the thalamic relay, which is consistent with previous studies (Scheck et al., 2016).

Measures of brain structure on both contralateral and ipsilateral hemispheres were retained in all models, regardless of the clinical outcome or the feature selection method used (Tables S1 and S2). Although many retained interaction effects were of ipsilateral regions where significant differences would be expected due to this hemisphere being uninjured in the unilateral cohort and injured in the bilateral cohort, several of retained interaction features were from the contralateral side, which is a more favorable outcome for motor performance than ipsilateral reorganization (de Almeida Carvalho Duarte et al., 2017). Although both models retained several areas on the cortex including the ipsilateral supplementary motor area, cingulate and paracingulate sulci, postcentral gyrus and contralateral precentral gyrus, there were only three interaction terms that were consistent across both AHA and MUUL models, which were the contralateral inferior frontal gyrus, contralateral middle temporal gyrus and ipsilateral precuneus. These regions are associated with language comprehension, facial recognition, and visuospatial memory respectively. Investigating cohort‐wise differences revealed increases in CT in these regions, with significantly increased thickness in the inferior frontal gyrus, particularly in the unilateral cohort compared to controls. Whereas brain injury would result in reduced volumes and CT, an increase CT in these regions may indicate potential neuroplastic mechanisms with the relocation of motor and sensory function to nearby regions in either hemisphere (Fiori et al., 2018; Simon‐Martinez et al., 2019), specifically the inferior frontal gyrus which lies anterior and inferior to the motor cortex, and the precuneus which lies posterior. Provided functional validation with fMRI confirmed plasticity of motor function, excitation, and enhancement of these areas through TMS performed as an intervention may present an opportunity to induce plasticity (Thickbroom, 2007), and hence recovery, in these participants.

Two methods of feature selection were used in this study: PCA, which retains features that explain the most variance in the feature space, and a data‐driven feature selection approach that selects features that are found to be most important in accurately estimating the motor function score. These two methods retained a substantially different set of features, with PCA tending to retain more global measures of brain structure, such as tissue volumes, instead of local features such as lesion volume or cortical shape in a specific region. As shown in Table 3, the more global features selected by PCA led to a smaller drop in the test set performance (compared to training set performance) for both AHA and MUUL models when compared to data‐driven feature selection, suggesting that these global set of features tended to produce more generalizable models. Furthermore, measures retained by PCA are independent of which clinical measure was used. However, while the global features retained by PCA tended to reflect the overall patterns of injury, largely tissue loss and CSF volume increases, measures of potential localized plasticity were only reflected by data‐driven models as these measures are optimized based on the association with a specific clinical function. Therefore, the choice of feature selection should be made based on the goal of the model—models for the prediction of multiple patient outcomes should focus on global features which can be identified using PCA, while investigations in structural brain differences resulting from plasticity should utilize data‐driven feature selection with careful selection of clinical score with which to optimize.

One limitation of this study is that only participants with a mild to moderate clinical presentation of hemiplegia were included in the study. Although this cohort included children and adolescents with severe brain injury that the automated processing methods must be robust to, participants with severe hemiplegia have increased difficulties in completing the imaging assessment and hence were not recruited. Consequently, the observed findings may not generalize to this group, and hence the random forest models would require validation on other cohorts to ensure generalizability. Another limitation is that handedness was not included in the regression models, hence definitions of ipsi‐ and contra‐lateral hemispheres defaulted to the left and right hemispheres. This assumes that these hemispheres were anatomically similar, which may not be the case for pediatric populations. Additionally, the differences in structure observed between cohorts may be due to differences in patterns of brain injury or neuroplastic compensation, which are not easily separated from structural imaging alone. Functional imaging using fMRI or TMS would be needed to demonstrate the functional relocation of hand function to other cortical areas, which has been investigated previously (Chen et al., 2019; Staudt, 2010), or alternatively longitudinal imaging would be needed to associate changes in brain structure with changes in patient outcomes.

Despite this, the results indicate that nondominant hand performance can be accurately predicted from structural MRI alone, as demonstrated by the significant test set correlations and low root mean square errors obtained on this cohort (Table 3). Furthermore, this was observed on an independent test set, indicating that these models could give insights into the global and local patterns of injury for other patients with uni‐ and bilateral brain injuries. To facilitate clinical translation of these model findings, a single decision tree representing the whole forest of trees in the model can be generated, where the important structural biomarkers are at each node, and split into either affected or unaffected by injury, and resulting in an average AHA and MUUL score at the leaves of the tree. This would allow clinicians to produce an estimate of AHA or MUUL from visually observation of the MRI. Furthermore, the local cortical features that were retained in these models may indicate potential areas of neuroplasticity if increases in regional GM volume were observed. As such, these findings may present a future therapeutic target, potentially with targeted interventions or TMS which have been shown to be effective therapies for neuromuscular impairments in spastic CP patients (Mehdinezhad, Taghiloo, Nourian, Nourian, & Mirbagheri, 2018; Parvin et al., 2018), to improve outcomes for children with bilateral brain injury.

## 5 CONCLUSION

Using pathology‐robust segmentation approaches and random forest regression, significant and generalizable associations between brain structure and hand function were observed in children and adolescents with unilateral CP, including those with bilateral brain injury. Regardless of the laterality of brain injury, significant correlations with two clinical motor scores were observed on an independent test set, indicating patient hand function can be accurately estimated from structural MRI data alone. More importantly, these approaches allow investigation of potential reorganization, particularly in cases where both hemispheres are affected by brain injury. Surprisingly, no difference in motor function were found between patients with unilateral and bilateral brain injury. The data‐driven prediction models revealed global reduction in brain volumes, including the DGM and corpus callosum splenium, and increased CSF and ventricle volumes, reflecting the greater impact of injury across the cerebral hemisphere. Local features associated with motor function were also identified in these models, including areas traditionally involved in motor function (internal capsule, thalamus, corpus callosum, precentral gyrus, supplementary motor area) as well as several cortical areas not associated with motor function (inferior frontal gyrus, precuneus), reflecting potential neuroplastic compensation in these cortical regions. In future, these areas may present interesting targets for therapeutic interventions, such as TMS, to improve outcomes of patients with UCP.

## Supporting information


**Table S1** List of retained features using data‐driven approaches for both AHA and MUUL models. Bolded features are common across both AHA and MUUL models.
**Table S2**. List of retained features using PCA for both AHA and MUUL models. Bolded features are common across both AHA and MUUL models.

## Data Availability

The data that support the findings of this study are available on request from the corresponding author. The data are not publicly available due to privacy or ethical restrictions.
