# Supplementary material for: Understanding the impact of bilateral brain injury in children with unilateral cerebral palsy
Source: Hum Brain Mapp. 2020 Mar 5;41(10):2794–807. doi: 10.1002/hbm.24978 (PMC7294067; doi:10.1002/hbm.24978)
Supplement: Supplementary file 1 — Table S1 List of retained features using data‐driven approaches for both AHA and MUUL models. Bolded features are common across both AHA and MUUL models. Table S2. List of retained features using PCA for both AHA and MUUL models. Bolded features are common across both AHA and MUUL models. [file HBM-41-2794-s001.docx]

# Supplementary Tables

Supplementary Table 1. List of retained features using data-driven approaches for both AHA and MUUL models. Bolded features are common across both AHA and MUUL models.

| *AHA* | | *MUUL* | |
| --- | --- | --- | --- |
| Feature | Feature importance | Feature | Feature importance |
| **Contralateral ALIC volume** | 0.256 | Contralateral thalamus volume | 0.170 |
| Contralateral PLIC volume | 0.030 | **Contralateral ALIC volume** | 0.054 |
| **CT of contralateral inferior frontal gyrus** | 0.030 | CT of contralateral precentral gyrus | 0.070 |
| CT of contralateral SMA | 0.029 | **CT of contralateral inferior frontal gyrus** | 0.066 |
| CT of contralateral lingual gyrus | 0.009 | CT of contralateral heschl gyrus | 0.015 |
| CT of contralateral superioroccipital gyrus | 0.016 | SD of contralateral superior frontal gyrus | 0.019 |
| SD of contralateral middle temporal gyrus | 0.011 | Curvature of contralateral precentral gyrus | 0.011 |
| Curvature of contralateral superior frontal gyrus | 0.010 | **Ipsilateral ventricle volume** | 0.063 |
| Curvature of contralateral cuneus | 0.010 | Ipsilateral cerebellar WM volume | 0.062 |
| **Ipsilateral ventricle volume** | 0.053 | CT of ipsilateral posterior cingulate gyrus | 0.005 |
| CT of ipsilateral cingulate and paracingulate gyri | 0.045 | SD of ipsilateral superior fontal gyrus | 0.013 |
| Curvature of ipsilateral calcarine fissure | 0.014 | Contralateral WM volume * Cohort | 0.065 |
| Contralateral ALIC volume * Cohort | 0.062 | CT of contralateral inferior frontal gyrus * Cohort | 0.021 |
| Contralateral PLIC volume * Cohort | 0.066 | CT of contralateral amygdala volume * Cohort | 0.009 |
| CT of contralateral olfactory cortex * Cohort | 0.013 | CT of contralateral supramarginal gyrus * Cohort | 0.019 |
| **CT of contralateral middle temporal gyrus * Cohort** | 0.027 | **CT of contralateral middle temporal gyrus * Cohort** | 0.060 |
| CT of ipsilateral SMA * Cohort | 0.018 | Curvature of contralateral hippocampus volume * Cohort | 0.036 |
| CT of ipsilateral superior frontal gyrus * Cohort | 0.093 | Curvature of contralateral fusiform gyrus * Cohort | 0.015 |
| CT of ipsilateral cingulate and paracingulate gyri * Cohort | 0.069 | CT of ipsilateral cuneus * Cohort | 0.033 |
| **CT of ipsilateral precuneus * Cohort** | 0.061 | CT of ipsilateral inferior occipital gyrus * Cohort | 0.029 |
| SD of ipsilateral olfactory cortex * Cohort | 0.059 | **CT of ipsilateral precuneus * Cohort** | 0.046 |
| SD of ipsilateral insula * Cortex | 0.013 | SD of ipsilateral superior frontal gyrus * Cohort | 0.016 |
| SD of ipsilateral superior temporal gyrus * Cortex | 0.006 | SD of ipsilateral superior occipital gyrus * Cohort | 0.019 |
|  |  | SD of ipsilateral middle occipital gyrus * Cohort | 0.040 |
|  |  | Curvature of ipsilateral calcarine fissure * Cohort | 0.019 |
|  |  | Curvature of ipsilateral postcentral gyrus * Cohort | 0.026 |
| Model R^2^ | 0.697 | Model R^2^ | 0.522 |

ALIC, Anterior Limb of the Internal Capsule; CT, Cortical Thickness; PLIC, Posterior Limb of the Internal Capsule; SD, Sulcal Depth; SMA, Supplementary Motor Area

Supplementary Table 2. List of retained features using PCA for both AHA and MUUL models. Bolded features are common across both AHA and MUUL models.

| *AHA* | | *MUUL* | |
| --- | --- | --- | --- |
| Feature | Feature importance | Feature | Feature importance |
| **Ipsilateral ventricle volume** | 0.256 | Age | 0.077 |
| Contralateral ventricle volume | 0.021 | **Ipsilateral ventricle volume** | 0.100 |
| **Ipsilateral GM volume * Cohort** | 0.043 | Ipsilateral WM volume | 0.013 |
| **Contralateral WM volume * Cohort** | 0.156 | **Ipsilateral GM volume * Cohort** | 0.013 |
| **Contralateral cerebellar GM volume * Cohort** | 0.010 | **Contralateral WM volume * Cohort** | 0.136 |
| **Contralateral WM volume** | 0.035 | **Contralateral cerebellar GM volume * Cohort** | 0.011 |
| **Contralateral GM volume** | 0.023 | **Contralateral WM volume** | 0.111 |
| **Contralateral thalamus volume** | 0.023 | **Contralateral thalamus volume** | 0.228 |
| **Splenium of the corpus callosum** | 0.007 | **Splenium of the corpus callosum** | 0.028 |
| **Contralateral PLIC volume * Cohort** | 0.259 | **Contralateral PLIC volume * Cohort** | 0.012 |
| Contralateral ventricle volume * Cohort | 0.018 | Contralateral cerebellar WM volume * Cohort | 0.023 |
| **Contralateral thalamus volume * Cohort** | 0.180 | **Contralateral thalamus volume * Cohort** | 0.203 |
|  |  | **Contralateral GM volume** | 0.045 |
| Model R^2^ | 0.570 | Model R^2^ | 0.480 |

ALIC, Anterior Limb of the Internal Capsule; GM, Grey Matter; PLIC, Posterior Limb of the Internal Capsule; WM, White Matter.
